# Stakeholders' Knowledge, Attitude, and Perceptions on the Control of *Taenia solium* in Kamuli and Hoima Districts, Uganda

**DOI:** 10.3389/fvets.2022.833721

**Published:** 2022-04-07

**Authors:** Nicholas Ngwili, Lian Thomas, Samuel Githigia, Nancy Johnson, Raphael Wahome, Kristina Roesel

**Affiliations:** ^1^Animal and Human Health Program, International Livestock Research Institute, Nairobi, Kenya; ^2^Faculty of Veterinary Medicine, University of Nairobi, Nairobi, Kenya; ^3^Institute of Infection, Veterinary and Ecological Sciences, University of Liverpool, Neston, United Kingdom; ^4^International Food Policy Research Institute IFPRI, Washington, DC, United States; ^5^Department of Veterinary Medicine, Institute of Parasitology and Tropical Veterinary Medicine, Freie Universität Berlin, Berlin, Germany

**Keywords:** *Taenia solium*, control strategies, knowledge, attitudes, perceptions

## Abstract

*Taenia (T.) solium* is a zoonotic parasite causing three diseases: Taeniasis and cysticercosis in humans and porcine cysticercosis in pigs. Although biomedically, the transmission of the parasite can be easily interrupted at six points along the life cycle, the contextual factors that may influence the adoption of these control strategies in Uganda remain unclear. This study assessed the stakeholders' knowledge, attitudes, and perceptions relating to the six control strategies for *T. solium* infections in Kamuli and Hoima districts, Uganda. A total of 22 focus group discussions (FGD) were conducted with pig farmers, community leaders, pig/pork traders, animal health assistants, and human health assistants. In addition, nine key informant interviews were held with senior officials in the ministries of agriculture and health and other relevant agencies at the district level. The results showed differential, limited, and fragmented knowledge on *T. solium* infections among stakeholders. Pig farmers, community leaders, and pig/pork traders had almost no knowledge and were often confused regarding the differences existing between *T. solium* and other gastro-intestinal infections in pigs and humans. Pig confinement, pit latrine construction, coverage, maintenance, and sustained use are influenced by cultural, socio-economic, and physical/ environmental factors of the study population and area. Proper sensitisation programmes and health education interventions should target all, but with appropriately focused material to suit the different stakeholder categories. Reminders or nudges may be needed to ensure that increase in knowledge translates to changes in practise. Intervention programmes should also aim to overcome challenges created by the various contextual factors operating in the specific endemic areas.

## Introduction

The local demand for pork has significantly driven growth in pig production in Uganda since the 1990's (1, 2). Around 70% of the pork produced in Uganda is consumed domestically at roadside butcheries and eateries, commonly known as pork joints (3). The majority of pigs are raised by smallholder farmers who are resource-constrained and rear pigs extensively with little investment in housing and feeding (4). Many of the pigs are either tethered or intermittently housed, depending on seasonality. They are fed mostly on crop residues (5). The rural areas in Uganda are also characterised by low coverage and underuse of sanitation facilities (6), creating a suitable environment for the transmission of *Taenia* (*T.) solium*.

The *T. Solium* is a zoonotic parasite causing three diseases: Taeniasis and cysticercosis in humans, and porcine cysticercosis in pigs. Taeniasis is the presence of adult tapeworms in the intestines of humans due to the consumption of undercooked pork containing viable cysts. In pigs, the ingestion of the tapeworm eggs from the environment leads to the development of cysticerci in the striated muscles, a condition known as porcine cysticercosis (PCC). Humans can also be infected by cysticercosis after ingestion of the tapeworm eggs shed by themselves or other humans. If the cysticerci lodge in the central nervous system, it leads to neurocysticercosis (NCC), a disease of serious health and social burden (7–9).

The transmission of the parasite can be interrupted at six points, along with the life cycle, as simplified in the “*Lets break the pork tapeworm cycle*” poster (10). These include: (1) use of toilets, (2) washing of hands, fruits, and vegetables, (3) regular deworming of children and adults, (4) pig confinement, (5) proper meat inspection, and (6) proper cooking of pork (10). In order to reduce the burden of NCC, three control strategies have also been proposed and tested for effectiveness at the community level in different endemic settings, including mass drug administration (MDA) of praziquantel to control taeniasis in humans (11), vaccination of pigs with TSOL18 vaccine combined with treatment using oxfendazole (12), and health education (13, 14). The success of the different control strategies may be influenced by contextual factors operating in target areas, including socio-economic, cultural, geographical, and environmental factors (15).

In Uganda, the socio-economic, cultural, and other factors that may influence the adoption of the six control strategies aimed at disrupting the transmission of the parasite have not been studied. The study, therefore, aimed to determine the knowledge, attitude, and perceptions of different stakeholders on the control of *T. solium* in the Kamuli and Hoima districts in Eastern and Western Uganda, respectively.

## Materials and Methods

### Ethical Statement

Ethical clearance was obtained from the International Livestock Research Institute's (ILRI) Institutional Research Ethics Committee (ILRI-IREC), reference number ILRI-IREC 2019-20 with extension reference number ILRI-IREC2019-20/2, respectively. Since the study was conducted in Uganda, approval was also obtained from the Research and Ethics Committee at the College of Veterinary Medicine, Animal Resources and Biosecurity, Makerere University (reference. SBLS/HDRC/19/008), along with a research permit obtained from the Uganda National Council for Science and Technology (reference A606). Before the start of the meetings, consent to participate and allow recording of the discussion was sought from both the focus group discussion (FGD) and key informant interview (KII) participants, and all of them signed an informed consent form. A consent form translated into the local language was explained to those unable to read and write, and they confirmed participation by inserting a thumbprint on the consent form.

### Study Area

**Figure 1:**
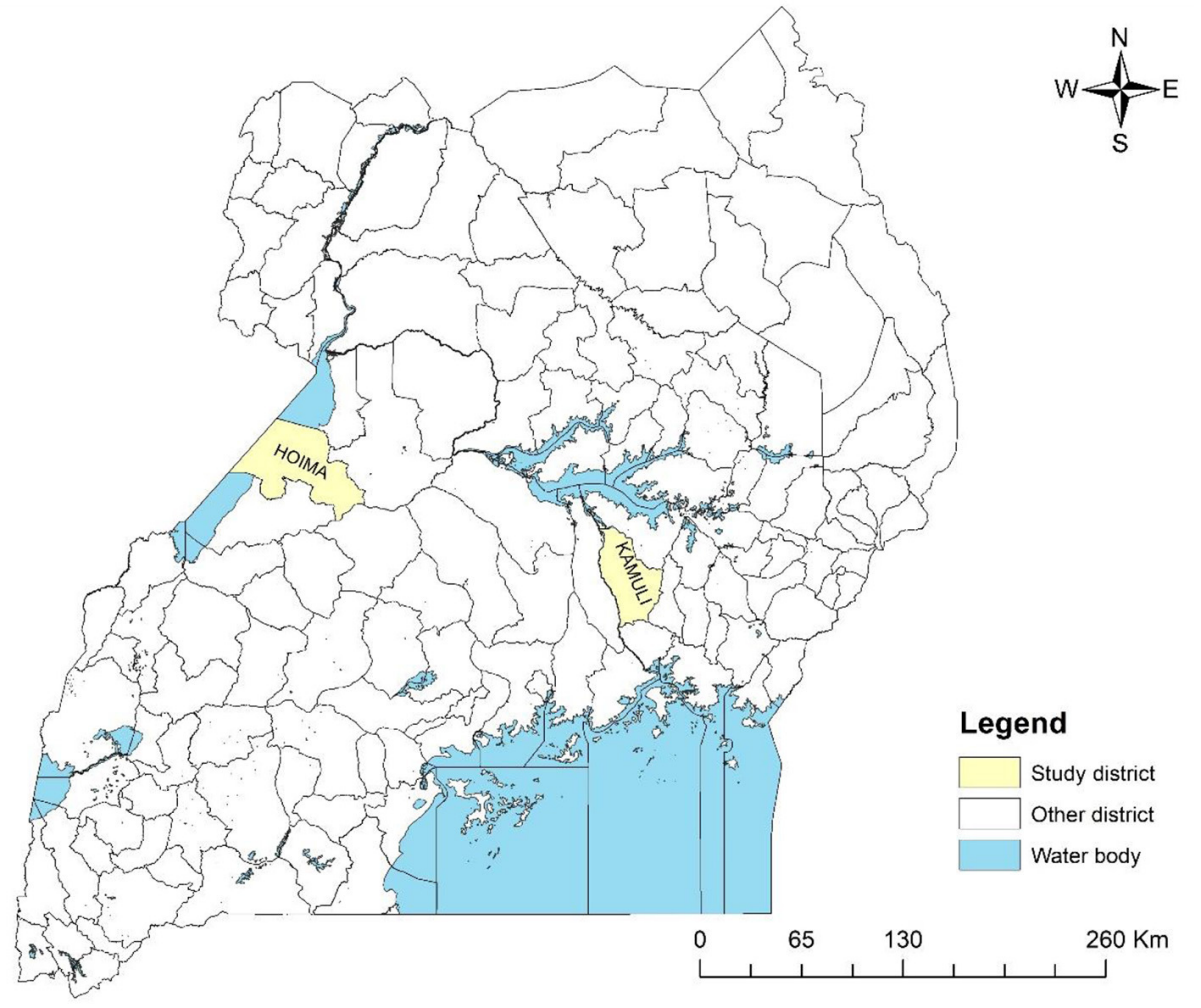
Map of Uganda showing the study districts (shaded in yellow).

The study was conducted between March and April 2021 in the Kamuli and Hoima districts, Uganda (Figure 1). These districts have high numbers of pig rearing households and high demand for pig meat and pig products Ouma et al. (16). The districts of Kamuli and Hoima were chosen because they have been sites for the International Livestock Research Institute (ILRI)-led research on the pig value chain. This made entry into the study site easy because local stakeholders had already established contact. The pig value chain of the districts had also been previously well-characterised Asiimwe et al. (17), Ouma et al. (2). In each district, community participants were drawn from villages within three sub-counties. However, the central government ministry official participants were drawn from across the districts.

### Study Design and Selection of Stakeholders

A community-based, qualitative study design was used. The FGDs comprising of 8–10 participants and KII were used to collect qualitative data. Different stakeholder categories play different roles in *T. solium* control. Therefore, the FGDs were organised by stakeholder category. The FGDs and KIIs were conducted as per the identified stakeholder categories shown in Table 1. To identify the stakeholders, a preliminary list was generated based on the pig value chain scoping visit conducted in 2014 under the smallholder pig value chain development project led by ILRI (18). The researcher then visited the two sites (the Kamuli and Hoima districts) to identify the specific stakeholders, explain the project, and check their availability to participate. For the pig farmer stakeholder category, separate FGDs were held for men and women. This was done to ease the collection of information across gendered toilet use and cleaning, maintenance, and pork preparation practises. Ten community leaders from each district were randomly selected from a list of 30 village leaders from villages that participated in an earlier cross-sectional study in 2019 (19). For farmers, the participants were randomly selected from a list of pig farmers who participated in the cross-sectional study in 2019 (19). The random function in excel was used for the randomisation. A maximum of 10 participants per category were invited for the FGD to ensure social distancing as per corona virus disease 2019 (COVID-19) pandemic protocols.

**Table 1:** Stakeholder categories targeted for data collection and their description.

**Stakeholder category**	**Description/target person or group**	**Relevance to *T. solium* control**	**Method for data collection**
Pig farmers	Pig farmers randomly from a list of pig farmers from 30 villages	They are responsible for control of the parasite at the intermediate and final host stage by practising proper hygiene and good pig husbandry.	FGD
Community leaders (LC1)	Selected randomly from villages across 3 sub-counties	They are village leaders and are the link between national government administration and community. They are involved in enforcing latrine use and other bylaws within the village.	FGD
Animal health assistants	Purposively invited through the District veterinary officer and were drawn from the different sub-counties in the district.	They oversee meat inspection and promotion of good animal husbandry at sub-county level.	FGD
Human health assistants	Purposively selected and invited through the District health officer and were drawn from the different sub-counties in the district.	They oversee human health activities in a sub-county and act as the heads of level 3 health facilities (the government health facility at the sub-county level	FGD
Pig/pork traders	Selected by snowballing from different sub-counties within the district starting from the district headquarters. Three traders were picked from each sub-county.	They buy pigs from farmers and operate butcheries and pork joints at the sub-country level where they sell raw and ready-to-eat pork.	FGD
District veterinary officers (DVO)	One officer from each district of study. Hoima district was recently subdivided in to 2 and therefore 2 DVOs were included.	They oversee veterinary and animal production in the district including meat inspection.	KII
District health officers (DHO)	One officer from each district of study (1 from Kamuli and 2 from Hoima district).	They oversee human health activities in the district including promotion of community hygiene.	KII
Private company (Devenish Nutrition in Hoima)	Outreach officer	The private company is involved in training of farmers and sale of inputs to pig farmers.	KII
Catholic NGO (HOCADEO-Hoima)	Veterinary extension officer	They are involved in promotion of pig husbandry and general household hygiene including toilet construction	KII
Neglected Tropical disease focal person under vector control division Ministry of Health–Kamuli	One official in Kamuli	They oversee mass drug administration campaigns in the district to control schistosomiasis. Praziquantel which is the drug of choice also treats taeniasis.	KII
National Animal Genetic Resources centre and Databank (NAGRIC & DB)	Head of community breeding programme	They are involved in extension work promoting improved pig husbandry.	KII
Iowa state university Uganda programme	The head of programme in Kamuli field office	They are involved in extension work promoting improved pig husbandry as well as household nutrition.	KII

### Data Collection and Management

**Figure 2:**
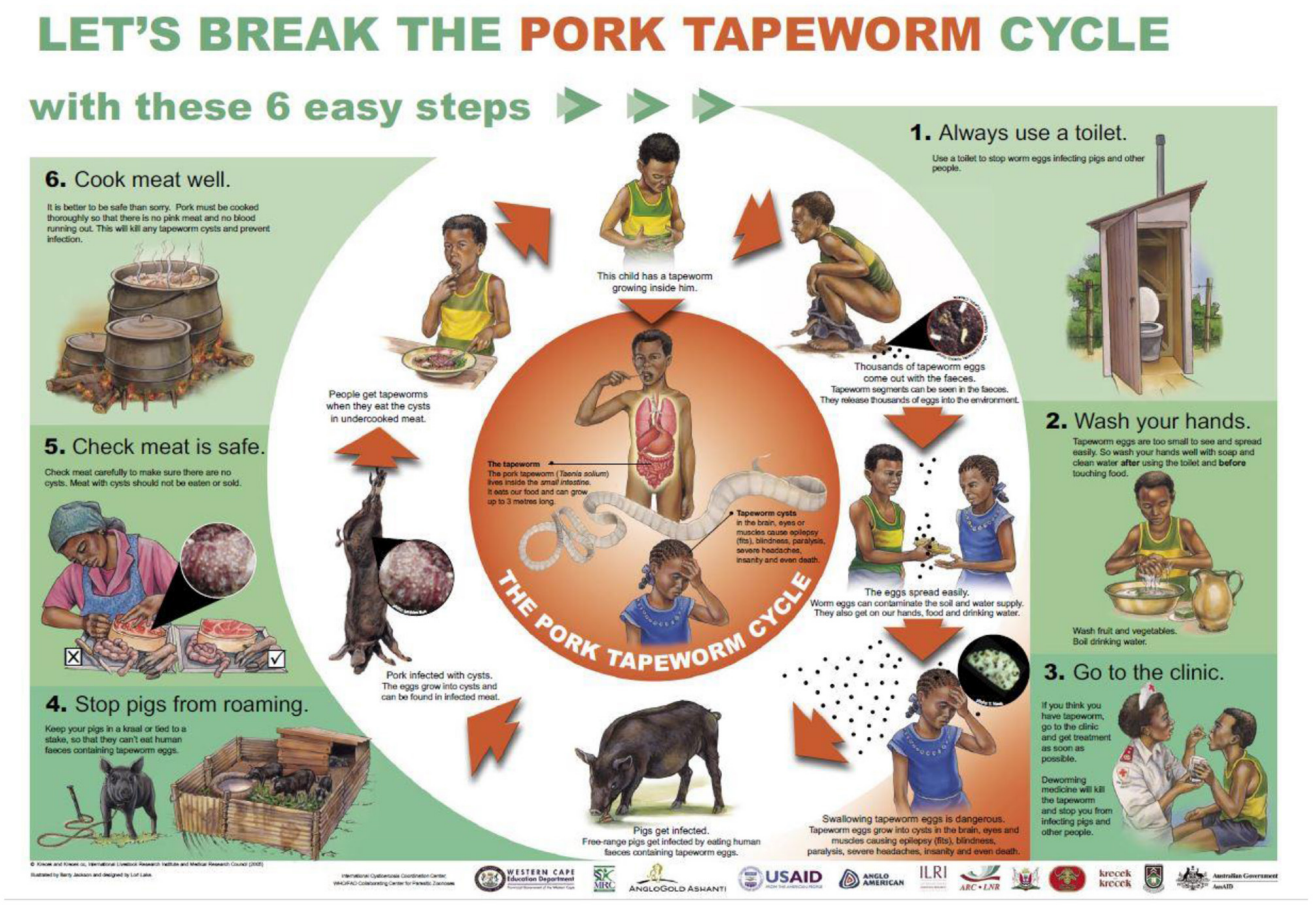
The “let's break the pork tapeworm life cycle” poster: source (10).

An FGD and KII checklist was developed, along the six points, where the transmission of *T. solium* can be interrupted in its life cycle as outlined in Figure 2. The FGD and KII guides were pretested in the peri-urban areas of Kampala with the different stakeholder categories. Changes were reviewed by the study team and adjustments were made to the guides.

Data were collected by 2 facilitators who were fluent in Runyoro/Runyankole (the local language in Hoima) and Lusoga (the local language in Kamuli), with one acting as a moderator and the other as a note-taker who captured non-verbal aspects of the discussion, i.e., hand counts, while supporting the moderator with timekeeping and in the case that some aspects of the guide were omitted. The FGDs with the ministry officials were conducted using both English and the local language understood by the participants. The KIIs were exclusively conducted in English by the lead researcher. All the FGDs and KIIs were audio-recorded using an electronic recorder. The audio files were transcribed verbatim into English by transcribers fluent in Runyoro/Runyankole and Lusoga languages. The typed scripts were verified by listening to audio files and comparing them with the notes. A coding frame was first developed by the lead author (NN) using prior knowledge on the control of *T. solium* based on the poster described earlier. The data were then coded into the respective codes or themes. The data were analysed using the deductive content analysis (20) and aided by NVIVO version 12 (21).

## Results

After a description of the demographic characteristics of the participants and their knowledge of *T. solium*, the results section is then divided into sections, along with the six points, where the transmission of *T. solium* infections can be interrupted.

### Demographic Characteristics

A total of 210 discussants participated in 22 FGDs that comprised of 12 FGDs with pig farmers, two FGDs each with animal health assistants, human health assistants, community leaders, and pig/pork traders. For the pig farmers, six FGDs were conducted in each district, and one FGD in each district for the other stakeholder categories. Nine more KIIs were conducted in both the Kamuli and Hoima districts, three with district veterinary officers, two with district health officers, one with a veterinary officer working for a local catholic relief organisation in Hoima, one each with a local private company in Hoima, the Neglected Tropical Diseases focal person under the vector control division of Ministry of Health in Kamuli, the head of the community breeding programme for National Animal Genetic Resources Centre and Data Bank (NAGRIC & DB), and the director of Iowa State University Uganda programme (https://www.globe.iastate.edu/global-experience/extension-projects-uganda/). Two FGDs were also conducted with leaders of local pig farmer associations in the Kamuli and Hoima districts. A full list of FGDs and KIIs can be found in Table 1. In total, 57 men and 59 women pig farmers attended the FGDs in both the Kamuli and Hoima districts. The demographic characteristics of the participants are presented in Table 2.

**Table 2:** Demographic characteristics of the study participant categories.

**Demographic characteristics (numbers)**		**Kamuli**	**Hoima**
Number of FGD	Men pig farmers	3	3
	Women pig farmers	3	3
	Animal health assistants	1	1
	Human health assistants	1	1
	Community leaders	1	1
	Pig/pork traders	1	1
Number of FGD participants	Men pig farmers	28	28
	Women pig farmers	29	31
	Total	57	59
Key informants (male and female)	Animal health assistants	10	8
	Human health assistants	10	10
	Community leaders	10	9
	Pig/pork traders	10	9
Pig farmers' level of education (%)	None	5.6%	0.0
	Primary	57.4%	45.8%
	Secondary	37.0%	44.1%
	Tertiary	0.0	10.2%
Pig farmers' mean age (in years)	Men	44.3	45.7
	Women	44.3	40.7
	Combined	44.3	43.2
Mean number of pigs		3.3	4.5

### Knowledge and Awareness on *Taenia solium* Infections

During the FGDs with the various stakeholders, it was established that there were differential levels of knowledge on *T. solium* and its control. Among the pig farmers, there was generally poor knowledge and awareness about the pork tapeworm. The majority of the farmers thought that the pork tapeworm is a type of worm infection that is found in the stomach or intestines of pigs, whereas the parasite manifests as small cysts within the musculature.

“*The tapeworm is found in the stomach [of pigs]; it affects the intestines of the pigs leading to stunted growth. The tapeworm is white and lives around the intestines” -* F10, women FGD, Hoima district.

Some of the farmers described the worm as being whitish. However, some participants were fully aware and had also seen the tapeworms in the faeces of children.

“*It manifests in humans. When defecating you can easily identify that a child has tapeworm after he has defecated”* – P3, Men FGD, Kamuli district.

Similarly, pig/pork traders and community leaders in both districts had poor knowledge on the pork tapeworm manifestation in pigs, although the majority agreed that pigs get infected when they roam around and feed on dirt, including soil, they considered it as a gastrointestinal worm.

“*Tapeworms are small, and they affect pigs if they are not treated” – R4* pig trader, Hoima district.

“*The tapeworm we are talking about is white in color and it looks like a round tapeworm, and it is elastic. When it is in an animal, especially the pigs, it makes it stunted, with swollen stomach”. -* P1, Community leaders, Kamuli district.

Many of the discussants in the animal and human health assistants' stakeholder category showed good knowledge of the *T. solium* infections, although some could not clearly link the tapeworm to human neurocysticercosis. The KIIs with the district veterinary and health officers showed that they had good knowledge and understanding of *T. solium* infection. The other four KIIs (local catholic relief organization veterinary officer in Hoima, local private company in Hoima, the Neglected Tropical Diseases focal person in Kamuli, and the head of community breeding programme for NAGRIC & DB) did not have comprehensive knowledge on the parasite, but they identified it as being a zoonotic parasite.

Pig farmers believed that pork tapeworm infections occur in pigs due to consumption of raw sweet potatoes, cassava peeling, or generally dirty and soiled feeds, and when the pig scavenges as it roams. Although farmers recognised that people acquired infections by eating foods or drinking water contaminated with faeces, there was confusion with the majority alluding that infection in humans can be through contact, sharing personal items or through the eggs penetrating the skin on the feet, implying threadworms. There was also a belief that it could arise naturally or automatically without any specific cause.

“*If an infected person defecates in the open environment, the feaces can be taken by running water to water sources and the egg if that water is taken un-boiled, the human being gets the tapeworms” – R9*, men FGD, Hoima district.

The above statement is wrong since ingestion of eggs results in human cysticercosis rather than tapeworm infection.

“*I think it's somebody's nature to have tapeworms […].. Whenever you deworm them, the tapeworms come out so it's his nature to have those tapeworms”. –* R5, Women FGD, Kamuli district.

The discussants in the animal and human health assistants' FGD had good knowledge and understanding of the infections in humans and pigs. The community leaders also had good knowledge of the infections, but they had poor knowledge of the route of infection.

“*Human beings get infected by tapeworms through eating unwashed raw fruits like mangoes and uncooked sweet potatoes. If a human being steps on the feaces of an infected person with the eggs of the tapeworm, they also get infected”* – R6, community leaders, Hoima district.

“*The pigs are the intermediate host. Human beings are the final hosts”* – R5, Animal health assistants, Hoima district.

### Latrine Construction, Coverage, and Use

Discussants in the farmers' stakeholder category estimated the pit latrine coverage (i.e., households having toilets in their homesteads) in their villages to be over half. However, the majority agreed that half were in bad condition–lacking complete walls, a door, or a roof. The estimate by the human health assistants who were promoters of community hygiene was not different. In both districts, the human health assistants estimated the coverage to be slightly above half, with less than half having permanent structures. The discussants in the community leaders' category from both districts gave the highest estimate of latrine coverage. The human health assistants noted that in both the districts, the sub-counties along flood-prone areas like the banks of river Nile in the Kamuli district and the shores of Lake Albert in the Hoima district had low latrine coverage due to occasional flooding. Those without latrines were reported to use their neigbours' latrines or use a polythene bag to later throw, especially those in urban areas, go with a hoe and dig a hole in the farm to defecate in, or defecate in banana and/or sugarcane plantations or in nearby bushes.

Most of the latrines were reported to be semi-permanent structures constructed using locally available materials such as tree logs and grass, while the permanent ones were constructed using commercial materials like cement, bricks, and iron sheets. According to the community leaders in both districts, half of the households had semi-permanent structures, of which some were without doors, walls, roofs, and had poorly constructed slabs that made them difficult to use. The design of the latrines was determined by the availability and cost of construction and construction materials.

“*2/10 households have permanent latrines, and 5/10 households have semi-permanent latrines while 3/10 have latrines without shelters [wall and roof]. Some households dig the pit and put the slab and do not put the shelter”* – R5, Community leaders FGD, Hoima district.

“*Most of them are made of grass, mud, poles, and reeds”* – R1, Human health assistants, Hoima district.

“*They are permanent and temporary, and these are made of cement, bricks, iron sheets, gravel, sand, tiles and pipes”* – R6, Human health assistants, Hoima district.

Several barriers to pit latrine construction were identified. Many participants cited the lack of resources to buy building materials as the main challenge in latrine construction. Other challenges included lack of construction equipment (hoes and spades), lack of space to construct the latrine, weak soils, rocky areas, which made digging hard, high-water table, especially along the flood plains, traditional norms, and customs that inhibited older generations from constructing latrines in the earlier years.

“*Most people lack resources such as money and the materials for the construction of latrines”* – R2, Men FGD, Hoima district.

Also, ignorance on the importance of having a latrine was cited by the participants. In Hoima, pig farmer discussants noted that they had formed a group to help them mobilise resources for buying materials, digging, and constructing pit latrines as a group.

“*Some soils are weak making them break so fast. The costs of constructing latrines are high. And some soils are rocky making it difficult to dig pits”* – R5, Human health assistants, Hoima district.

For the semi-permanent latrines that were made using non-commercial materials, the role of men was to dig the pit, cut logs, and build the latrine. On the other hand, women supported the construction by cutting grass for thatching and fetching water. In terms of maintenance, most of the farmers agreed that women cleaned the latrines using brooms and ashes, trained the children on pit latrine use, and enforced latrine use. On the other hand, it was noted that, in most cases, the household head funded the construction of permanent latrines and paid for the mason.

At the community level, enforcement of pit latrine construction and promotion of household hygiene was reported to be carried out by the community leaders and village health teams (VHTs). The VHTs are village-based structures whose members are selected through a popular vote by the community members to promote their health and wellbeing. One member is selected from 25 to 30 households and is supported by the ministry of health, Uganda (22). Overall, this role was identified by discussants in the farmers, community leaders, traders, and human and animal health assistants' stakeholder category. This was also supported by the KIIs with the district health officers in both districts.

“*The village health teams [VHTs] and LC1[community leaders] do enforce the use of latrines in the villages. The LC1 work hand in hand to teach people about the importance of using a latrine”* – F8, Women FGD, Hoima district.

### Barriers to Latrine Use

Several barriers to latrine use were cited by the majority of the discussants from the farmers, community leaders, traders, and human and animal health assistants' stakeholder category. The barriers included:

(i) Age: Children below 5 years and the elderly, e.g., people above 65 years (23), did not use pit latrines as identified by the majority of farmers and community leaders. The children defecate around the latrine and the faeces were thrown in the latrine or the garden. The elderly dug a hole in the garden and defecate or defecate in bushes or sugarcane plantations. The toilets are mostly pit latrines that require users to squat and, thus, handicap the elderly. Those rendered weak by ill health were reported not to use them.
(ii) Poorly constructed latrines with weak slabs or openings that made people fear falling into the pit, as identified by some farmers and the community leaders.
(iii) Poor lighting in the latrines prevented their use at night out of fear of rodents and snakes.
(iv) Poor state of hygiene and crowding in public latrines during public functions or market days.
(v) Smelly latrines.
(vi) Wrong intentions for construction of latrines, where some construct latrines to be seen by enforcement officers to avoid punishment. Additionally, others constructed the wall and roof without the pit just to trick enforcement officers.
(vii) Cost minimisation led to fear of using the toilet to avoid getting it full and having to construct another one.
(viii) Drunkards and mentally disabled people were reported not to use latrines.
(ix) Beliefs such as:
  a. Women should not use latrines. Otherwise, they will never bear children;
  b. Pregnant women should not use latrines;
  c. Children's feaces should not be thrown in latrines as they are not harmful and will decompose when thrown in the garden.

### Handwashing and Personal Hygiene

The majority of the discussants in the farmer's and community leaders' stakeholder category noted that handwashing facilities were available near the latrine or in the compounds, usually in form of a foot-operated, small jerry-can (“tippy tap”), and in some cases, with available soap. It was also pointed out that this had become more common and adopted due to the ongoing campaign occasioned by the current COVID-19 pandemic.

“*People have learnt to have these jerry-cans for washing hands because of the COVID-19 outbreak but way back people never mind having a handwashing facility”* – P3. Men FGD, Kamuli district.

Although most households were reported to have had handwashing facilities, the community leaders noted that few people washed their hands after using the latrines. The community leaders and VHTs were involved in promoting and sensitising households on good hygiene, including the use of latrines and having a handwashing facility. The KIIs with district health officers pointed out that control of *T. solium* infection can be achieved by ensuring proper sanitation, including handwashing, in households, but the practise is not widespread.

“*When you are moving around, you find handwashing facilities. 7/10 households have handwashing facilities but only 2/10 households wash their hands after using the latrine*”- R5, Community leaders FGD, Hoima district.

### Deworming of Children and Other Household Members

Discussants amongst the farmers' stakeholder category had different views on when themselves or children should be dewormed, with some indicating that deworming should happen every 2, 3, or 4 months or once a year. The majority reported that they dewormed using ketrax tablets (levamisole), albendazole, or mebendazole, with a few using local herbs. The deworming drugs were either bought at a local drug shop, private clinic, or issued for free at government health facilities, especially to expectant women during the normal antenatal visits. The majority of the different stakeholders reported that there were no government deworming programs targeting the general population. Despite this, in some of the sub-counties, there were school deworming programs for school-age children (SAC). The majority of the discussants belonging to different stakeholder categories were aware of the existence of mass deworming programs targeting SAC to treat soil-transmitted helminths (STHs). Some of the discussants from the farmers' stakeholder category noted that they did not know that adults can get worm infections and thought that it is only a problem in children.

“*I did not know that even an adult person deworms, I knew children alone deworm. Government deworms children below five years”* – R1, Men FGD, Hoima district.

However, it was noted that expectant mothers were issued with deworming drugs during routine antenatal visits to the government health facilities. The human health assistants also noted that school health days were organised twice a year in both districts to promote children's health and deworming. According to the community leaders, deworming of children was also done during routine immunisation campaigns in the communities. The Iowa State University Uganda program had clinic days, where they invited local health centres to sensitise the general community on good nutrition, during which deworming was also offered as reported by the KI interview with the director of the programme.

### Confinement of Pigs

Pig farmers appeared to have knowledge that housing pigs has benefits, including prevention of diseases like African swine fever (ASF) and worm infections, and avoiding conflict with neighbours if the pigs roam into their farms. However, most of the farmers and community leaders noted that there were free-roaming pigs in their respective villages. Some farmers also confined pigs during the rainy season and let them roam during the dry season. The reasons for not housing pigs included: (i) lack of resources to construct pig pens, (ii) weak structures that were easily broken down by the pigs (iii) insufficient time available for their owners to care for housed pigs and attend to other business, thus, pigs were left on their own to roam and forage for feed, and (iv) pig feeds were also reported to be expensive, therefore, farmers preferred to leave the pigs to scavenge for feeds. Additionally, some farmers believed that free-roaming pigs grew faster as compared to confined pigs.

The animal health assistant discussants noted that the adoption of improved pig husbandry by farmers, including pig confinement, was moderate. They noted that the farmers put up simple structures because they had not taken pig rearing as a business venture. Despite this, some who had been trained and have been exposed to improved pig husbandry had good pig pens. The discussants also noted that farmers are discouraged from investing in pig housing because there are no price incentives for fat and well-reared pigs. The middlemen and traders preferred extensively reared small pigs because they obtained them at lower prices from the farmers. Moreover, because of low pork meat, traders buy small pigs that they can sell within 1–2 days due to the demand and lack of refrigeration in the rural areas.

“*The market dynamics have discouraged farmers from adopting good husbandry. The middlemen always prefer cheaper pigs than the expensive ones*” – R5, Animal health assistants, Hoima district.

### Meat Inspection

The majority of the discussants in the farmers' stakeholder category noted that inspection in the villages was not regular and was usually majorly conducted by a government official during holiday seasons, such as Christmas, when a lot of pigs were slaughtered. The majority of the farmers also noted that as consumers, they did not check meat for cysts because they did not know how to or what to check. The farmers also noted that the traders do not allow consumers to inspect the meat by touching it. When buying raw pork, they only check for the colour, amount of fat, the freshness of the meat, whether it is from a male or female pig (meat from female pigs is preferred because it was considered soft), and the general cleanliness of the butcher. The majority of the traders did not inspect for cysts when buying the pigs because they did not know how to check for cysts, but they checked for signs of ASF and mange infections in live pigs.

“*The responsible people [government official] don't inspect meat during the other normal days but rather they come during holidays when they know they are going to get a lot of money collections”* – R1, Women FGD, Hoima district.

The traders relied on the government meat inspectors, who at times failed to reach their slaughter place, for meat inspection. In those cases, they would go ahead and sell uninspected pork, but it was reported by one of the traders that while local consumers do not demand to see a meat inspection stamp, consumers from Kampala do. The discussants also noted that if the meat inspectors arrived late, they inspected the meat while it is already in the butchery being sold. Many of the discussants of the animal health assistants/meat inspectors' stakeholder category noted that there is a lack of centralised slaughter facilities that exposed them to harassment by disgruntled butchers if they condemned carcasses during the inspection. Carcasses were, therefore, rarely condemned. Meat inspectors instead reported that they issue stamps with conditions that meat is properly cooked or only condemned the infected part of the carcass.

“*[…]some of our customers from Kampala ask for the meat inspection stamp, we do not wait for him [meat inspector] we go ahead and sell uninspected pork. Some veterinary doctors come late, we sell and when she comes, she inspects as we are selling”* – R1, Trader FGD, Hoima district.

“*We lack facilities to do that, and we are not protected. Not only that there is no disposal site even if it is cattle carcass. So ideally, we lack are no facilities to burn it and even if you condemn, the meat still comes back to the market.”* – R1 Animal health assistants FGD, Kamuli District.

There was also political interference reported in terms of enforcement of the meat inspection laws. For instance, traders used political influence to prevent meat inspection from being conducted and evade enforcement. The participants in the KIIs with district veterinary officers (DVOs) noted that meat inspection is covered under the public health act of Uganda, but enforcement was constrained by lack of resources for transport to slaughter places, understaffing, lack of centralised slaughter facility, and political interference.

### Pork Preparation

The majority of farmers identified butcheries and pork joints as being the main sources of raw and cooked (ready to eat) pork. In some rare circumstances, households were reported to have bought a pig to be slaughtered and shared, especially during festivities. It was also reported that women were mainly responsible for preparing pork at home for consumption by household members.

Preparation of pork meat for consumption was done in several ways as was identified by the pig farmer FGDs discussants: (i) boiling to remove excess fat, adding ingredients like onions and tomatoes before frying; (ii) Roasting over wire mesh, cutting into small pieces, adding of ingredients, and frying until it is soft; and (iii) frying until it is well-cooked as indicated by a change in colour from white to brown. The majority of the discussants in the farmers' category reported that the barriers to cooking pork meat well at the household level included lack of enough firewood, impatience while cooking, lack of sufficient time to properly cook the meat, lack of awareness on the consequences of eating under-cooked pork, and the preference for under-cooked pork. When the pork was consumed in the pork joints, the consumers relied on the butchers to tell when pork is well-cooked or roasted.

Most of the discussants in the pork traders' stakeholder category noted that the barriers for them to cooking or roasting pork in their pork joints included: (i) lack of firewood for cooking/roasting, (ii) too many orders from customers, (iii) lack of awareness on the consequences of eating under-cooked pork, (iv) lack of roasting or cooking skills, (v) lack of utensils for cooking/frying like saucepans, and (vi) the preference of some customers for under-cooked pork. The majority of the discussants in the pig farmers' stakeholder category noted that eating poorly cooked pork could lead to vomiting, stomach pain, and diarrhoea. In the Kamuli district, it was noted that the consumption of raw pork leads to swollen cheeks. In the Hoima district, a few of the discussants said it led to brucellosis. None of the discussants among the pig farmers' category mentioned that it could result in infection with pork tapeworm.

## Discussion

Among the various type of stakeholders targeted, pig farmers, community members, and traders' categories had the lowest level of knowledge, specifically on *T. solium* infections. Similar findings were reported in Northern Uganda (24). There was a confusion of the pork tapeworm with other pig gastrointestinal helminths, with results similar to those reported in Eastern Zambia (25). This could be due to how farmers could easily identify infection of pigs with worms through physical symptoms such as stunted growth, reduced weight gain, emaciation, and identification of the nematodes in pig faeces. Pig gastrointestinal parasites are prevalent in Uganda and have been extensively reported in various locations including in the Kamuli and Hoima districts (19, 26, 27).

A limited number of participants were aware of tapeworm infection in children but not in adults. Taeniasis could be due to infections with either *T. solium* or *T. saginata*, neither of which have been well-studied in human populations in Uganda. Only one study reported a prevalence of 0.7% for taeniasis among school children in Kampala (28). As the participants could not clearly describe the worms seen in the faeces, they could have been other intestinal helminths reported in school-going children in Uganda (28, 29). Knowledge on the tapeworm was highest among human and animal health professionals albeit with confusion on how the infection with *T. solium* leads to neurocysticercosis. Similar findings were reported in Tanzania among veterinary extension officers and medical health professionals (30).

The infection of pigs with *T. solium* cysticercosis does not produce any identifiable clinical signs and may persist unnoticed in pigs. However, in contrast with findings of the current study, Kungu et al. (31), using a household survey, reported a high knowledge performance score of farmers on *T. solium* infection transmission in Eastern and Western Uganda. On the other hand, low knowledge levels on *T. solium* transmission in the general population have been reported in Tanzania (32). One limitation of these studies is that they used a “yes/no” knowledge question implanted in a household survey that may have not brought out the true underlying knowledge levels. Low awareness and knowledge on *T. solium* infections and transmission reported in this study may be a barrier to the adoption of practises aimed at breaking the transmission cycle and reducing the incidence and prevalence of the infections.

Although there was reportedly a relatively high pit latrine coverage in the study districts, many of the toilets were poorly constructed. The national latrine coverage in Uganda stood at 79% in 2018, with 3 out of 10 households lacking a latrine (33, 34). The high cost of toilet construction may have led to the construction of low-quality latrines with weak slabs or ones with large spaces between the poles on the floor, incomplete walls, or roofs. Latrine construction was also affected by the state of the ground, e.g., rocky, loose, or sandy soils, and high-water tables in areas along the flood plains, making it difficult for construction. Similar challenges due to soil formations were reported in Ghana (35). Günther et al. (36) noted that lack of money was the major barrier to investment in latrine construction in Uganda. The cost of constructing a ventilated pit latrine with a plastered brick structure was estimated at USD 760 in peri-urban Kampala (37). The median monthly wage for the rural population in Uganda was estimated at UGX 120,000, approximately USD 33 (at USD 1 = UGX 3,600), and UGX 220,000, approximately USD 61, for the urban population in 2016 (38). This may mean that majority of households may struggle or may be unable to construct a modern toilet given the estimated cost with this income level.

During latrine construction, men and women played different roles, with men taking up more physical activities like digging the pit, while women supported construction by fetching water and thatching materials. Nunbogu et al. (35) made similar observations in Ghana. Additionally, women were responsible for toilet maintenance, cleanliness, and latrine use enforcement. Dissemination of information and enforcement of latrine construction and use without capital investments may not be sufficient to increase coverage and sustained use. Furthermore, gendered roles on latrine construction use and maintenance should be considered when designing interventions to increase pit latrine coverage and use.

Although relatively high latrine coverage was reported in the current study, as was also estimated by the government of Uganda at 79% (33), open defecation, which is a risk factor for *T. solium* cysticercosis and other infections, was still reportedly practised, especially by the elderly, children, and, in some instances, other household members. Similar findings were reported in a systematic literature review on latrine coverage and use by Garn et al. (39), who noted open defecation even among households with latrines.

Open defecation in gardens can contaminate fruits, vegetables, and cassava or sweet potato tubers, presenting a risk for neurocysticercosis to household members. Some barriers to latrine use that promote open defecation included poor latrine design, poor access paths, poor lighting, and a low state of maintenance and hygiene. The first barrier did not guarantee privacy and ease of use while others discouraged use. These findings are consistent with findings by Kwiringira et al. (40), who reported that open defecation was practised in the slums of Kampala, Uganda and in Lodwar town, Kenya (41). Similarly, Exum et al. (42) reported that open defection in bushes or near water bodies was practised in different regions across Uganda. Failure to maintain the cleanliness of the pit latrine was found to be a significant factor contributing to the descent from the sanitation ladder back to open defecation in Uganda (40). On privacy during latrine use, Nunbogu et al. (35) reported that in Ghana, the assurance of privacy increased latrine usage by 42.5%.

Handwashing facilities were reported to be common in most households, but their use after visiting the toilet was considered by study participants to be limited in agreeance with Byamukama (43), who reported that the practise of handwashing after using the toilet was low in Uganda (52%), with only 14% using soap. A lack of handwashing and poor personal hygiene presents a risk of infections with *T. solium* cysticercosis to tapeworm carriers through the direct ingestion of eggs or to other household members through contamination of food and/or water. In a review on the availability of handwashing facilities in East African countries, using demographic health surveys, Kisaakye et al. (44) noted that Uganda had the least availability at 59.2%. The promotion of handwashing and improved personal hygiene is done by community leaders and VHTs, but may have not been achieving the desired impact. One recent intervention that has increased the awareness and the practise of handwashing is the promotion of the use of the tippy tap, which consists of a jerry can, a string, and a piece of wood in a lever system. It is operated by foot and, hence, avoids contamination of the handwashing facility (45).

The results of this study indicate that there was a positive attitude towards deworming, especially in children, but the practise is not common. There was low awareness on whether adults need to regularly deworm, with few discussants noting that they do deworm occasionally. There was no consensus on the frequency of deworming among the discussants. The WHO guidelines on preventive chemotherapy recommend annual or biannual deworming with single-dose albendazole (400 mg) or mebendazole (500 mg) in young children above 1 year, SAC, non-pregnant adolescent girls, and pregnant women after their first trimester (46). These guidelines are followed in Uganda (29). Deworming can break the *T. solium* transmission cycle by killing the adult tapeworms in humans and preventing environmental contamination. The commonly used and available deworming drugs in Uganda are Albendazole, which requires a 3-day regimen for the successful treatment of taeniasis (47, 48), and mebendazole for the treatment of *Enterobius vermicularis* (threadworms, also called pinworms), *Strongyloides stercoralis* (threadworm), *Trichuris trichiura* (whipworms), *Ascaris lumbricoides* (roundworm), *Necator americanus* (hookworm), and *Ancylostoma duodenale* (hookworm) (49). Triple dose mebendazole is also effective against taeniasis (50). It was noted that SAC was annually dewormed in school and during child's healthy days using praziquantel to control schistosomiasis. A single dose of praziquantel at 10 mg/kg is effective against *T. solium* taeniasis (51) and is the recommended drug of choice (52). The effect of the MDA campaign on the prevalence of taeniasis and incidences of *T. solium* cysticercosis in Uganda needs to be evaluated as was done in Tanzania (53).

Pig farmers had good knowledge and awareness of the importance of pig confinement in the control of diseases but keeping pigs on the free-range was still practised. There were also misconceptions and beliefs on pig confinement, with the belief that confined pigs do not grow as well as confined pigs. This may be true if the latter are poorly fed (5). Efforts to improve the adoption of pig confinement should also consider the barriers faced by farmers, including the availability of resources to construct pig pens and to buy feeds for the confined pigs and the lack of price incentives for properly raised pigs. Similar findings on barriers to pig confinement were reported in Zambia (25). An option could be to promote simple pig pen designs that could be constructed using locally available materials and alternative, more accessible feeds for pigs, such as forage and silage-based diets. These types of feeds were shown to reduce cost and have relatively good average daily gain (ADG) (54). In a study in Kenya, Levy et al. (55) concluded that small-scale traders, who could feed non-commercial feeds to pigs to attain a high ADG and could bargain with traders for better prices, were likely to benefit from semi-intensive pig farming. Low-cost, locally available, and nutritionally complete diets have also been formulated for pigs in western Kenya (56). Additionally, the traditional pig rearing sector was shown to be more sustainable than the intensive pig rearing system (57). Kabululu et al. (58) noted an improvement in pig confinement after an intervention that trained farmers through a demonstration on the construction of an improved pig pen and pig feed formulation. Results from the current study also show that pig traders demand smaller pigs (lower weight) due to the lower uptake of pig meat in rural areas and, possibly, because they lack refrigeration services and would have to sell the entire carcass in 1 or 2 days. Pig farmers in Uganda reared pigs as a form of saving, particularly to be sold for cash to cover school fees or emergencies (2). To ensure profitability for the enterprise by selling the pigs at the specified time or when a certain weight is attained, farmers may need alternative financial products to provide cash to cover emergencies and other household expenditures.

Meat inspection by government officials was reported to be irregular in the rural villages, only being conducted during holiday months when many pigs are slaughtered. Meat inspection of pigs slaughtered by the butchers across the district was reported to be irregular and ineffective due to the lack of a centralised slaughter place, lack of transport for the meat inspectors, and political interference. Thys et al. (25) reported similar challenges to meat inspection in Zambia. The traders did not mind if the carcass was not inspected. They went ahead and sold to buyers unless an inspection stamp was demanded, as was sometimes the case for Kampala consumers. Local consumers only checked meat for physical quality attributes and not for infections like cysts. These findings were similar to Roesel et al. (3), who reported in detail the attributes consumers in Uganda consider before buying both raw and/or ready to eat pork, including cleanliness, moderate fat layer, freshness, colour, texture, and smell of the meat in order of importance.

On the other hand, traders did not report inspecting pigs for porcine cysticercosis before buying. Instead, they checked for signs of ASF and external parasites in live pigs, whilst Ouma et al. (59) reported that traders inspected pigs for *T. solium* cysticercosis through tongue palpation in the Masaka and Bukedea districts, Uganda. This contrast may be because the study focused on districts, where traders buy pigs and transport them to Kampala, while, in the current study, the traders majorly bought and slaughtered for local consumption. The motivation to inspect for ASF may be due to fear of spreading the infections that may lead to market closure and animal movement restrictions that may adversely affect their businesses. This shows that the priority for traders is the effects that diseases can inflict on their business, but not necessarily on the risk of contracting zoonotic diseases through consumption of uninspected meat. The failure of the meat inspection system in the study area may mean that pork consumers are at risk of infection with taeniasis and, consequently, neurocysticercosis. Roesel et al. (60) also reported challenges in meat inspection and law enforcement in an analysis of Wambizzi slaughterhouses in Kampala, including illegal slaughtering before meat inspectors reported to work to avoid paying the slaughtering fee.

Stomach upsets, vomiting, and diarrhoea were reported as the main effects of consuming half-cooked pork. Generally, there were low knowledge levels and awareness on the risk of getting taeniasis by eating half-cooked pork. Households practised different methods of preparing pork at home, similar to findings by Roesel et al. (3). Health education with messages on cooking/roasting coupled with enforcement of standards on the sale of ready-to-eat food may be needed to lower the risk of exposure of consumers to infective meat.

### Limitations of the Study

The focus group discussants were selected from a list of farmers who had earlier participated in a cross-sectional study on risk factors for *T. solium* infections. This may have biassed the results due to the existence of prior knowledge gained from being involved in the earlier study. However, the current study focused more on aspects of control of the parasite which were missing in the earlier cross-sectional study.

## Conclusion

Pig farmers, community leaders, and pig/pork traders had almost no knowledge of *T. solium* infections and were often confused regarding the differences existing between *T. solium* and other gastro-intestinal infections in pigs and humans. Pig confinement, pit latrine construction, coverage, maintenance, and sustained use were influenced by cultural, socio-economic, and physical/ environmental factors of the study population and area. Proper sensitisation programmes and health education interventions should target all, but with material appropriately focused to suit the stakeholder category. Reminders or nudges may be needed to ensure that any increase in knowledge translates to changes in practise. Intervention programmes should also aim to overcome challenges created by the various contextual factors operating in specific areas. Additionally, adoption of the various practises to control *T. solium* require behavioural modification by the different stakeholders, participatory design of the intervention, and integrated behavioural change frameworks should be considered in the implementation of intervention.

## Data Availability Statement

The original contributions presented in the study are included in the article/Supplementary Material, further inquiries can be directed to the corresponding author.

## Ethics Statement

The studies involving human participants were reviewed and approved by ILRI Institutional Research Ethics Committee and Research and Ethics Committee at the College of Veterinary Medicine, Animal Resources and Biosecurity, Makerere University. The patients/participants provided their written informed consent to participate in this study.

## Author Contributions

NN, NJ, KR, and LT: conceptualisation. NN, RW, SG, NJ, KR, and LT: methodology. NN: data collection and writing–original draft. NN, KR, and LT: analysis and data interpretation. All authors: writing–reviewing and editing. All authors contributed to the article and approved the submitted version.

## Funding

The field research was funded by the Consultative Group for International Agricultural Research (CGIAR) Research Program on Agriculture for Nutrition and Health (A4NH) led by the International Food Policy Research Institute (IFPRI) and the German Academic Exchange Service (DAAD) through an in-region PhD fellowship in partnership with the International Livestock Research Institute (ILRI) awarded to NN (Grant No.91635410). KR and LT are supported by the BMZ One Health Research Education and Outreach Centre in Africa (OHRECA). Additionally, LT was supported by the University of Liverpool- Wellcome Trust Institutional Strategic Support Fund and the Soulsby Foundation (https://soulsbyfoundation.org/).

## Conflict of Interest

The authors declare that the research was conducted in the absence of any commercial or financial relationships that could be construed as a potential conflict of interest.

## Publisher's Note

All claims expressed in this article are solely those of the authors and do not necessarily represent those of their affiliated organizations, or those of the publisher, the editors and the reviewers. Any product that may be evaluated in this article, or claim that may be made by its manufacturer, is not guaranteed or endorsed by the publisher.
