# Supplementary material for: Stakeholders' Knowledge, Attitude, and Perceptions on the Control of Taenia solium in Kamuli and Hoima Districts, Uganda
Source: Front Vet Sci. 2022 Apr 7;9:833721. doi: 10.3389/fvets.2022.833721 (PMC9021822; doi:10.3389/fvets.2022.833721)
Supplement: Supplementary file 1 [file Data_Sheet_1.docx]

**Workshop guide for farmers/community members (group discussion) – probes have been included in brackets**

1. What is *Taenia solium/the pork tapeworm? (use local language for tapeworm)*

**probes**

1. perceived causes, how do pigs get infected,
2. how do humans get infected (probe for Taeniasis and NCC?
3. How the diseases manifests in humans and pigs,
4. use the *T. solium* poster to explain the three diseases and how they are acquired – Taeniasis, NCC and PCC)
5. Give overall results from the cross-sectional study (prevalence, risk factors).

**Probes**

1. What does it mean when cysts are found in pig meat?
2. people/children pass worms pieces in faeces) and how they can be used?
3. when you eat undercooked meat with cysts,
4. what disease can you end up getting
5. How can we control the tape worm infections (allow them brainstorm and use the poster to show possible control options (the 6 stages to break the cycle)?

**probes**

1. what can a mother/farmer/butcher do to break the cycle? -
2. Give an overview of the control options which can be done at community level and discuss their roles as outline below
3. Mass drug administration (MDA) in humans -PZQ (In general population or in School Age Children (SAC) – when children or adults are given deworming tablets by some NGOs or the government. **What can motivate you to participate in this option (deworming for other soil transmitted helminths?)**
4. Vaccine + oxfendazole (OXF) in pigs-*(vaccine-TSOL18 kills cysts in pigs, OXF kills T. solium cysts and kills other pig worms)* (will you be willing to pay for the vaccine? **What can motivate you to adopt this option? Is worm infection in pigs a problem in this** **area? Will use of a drug that kills both pig worms and *pork tapeworm* in pigs be suitable for you? why?**
5. Health education – on pig husbandry and household and community hygiene – use the T. solium poster (**why confining pigs and feeding them clean feed is important, why use of toilet and washing hands is important**

Do all the households in your village have toilets

**Probes**

percentage of households with toilets,

are the toilets well-constructed?

what is your role in construction of toilets?

who cleans the toilets,

Who enforces the use of toilet in your home and in your village?

Are there programs by government or NGOs supporting construction of toilets or hygienic infrastructure?)

**probes**

Where is there is latrine, which materials did people use,

who taught them how to build it,

do they like the design, if not, what do they not like,

do children use the toilet?

How do they empty and maintain them?

do they have hand washing areas next to the toilets?

1. Where do households without toilets relieve themselves? (**Probe for households with children below five years, elderly, men and women**)
2. Are there households with toilets and do not use them? (**Probe for what makes people not to use toilets even if they are available)**
3. What are the constraints to toilet construction?

**Probe**s

1. How do the constraints affect men and women?
2. what can be done to increase construction and use of toilets
3. Do the local marketplaces have a public toilet (**Probe to know where people relieve themselves while in the local markets, during village gathering/meetings)**
4. Are there free roaming pigs in your village?

**Probes**

1. What do they feed on when scavenging?
2. Why the pigs not housed?
3. What are the consequences of let pig roam around?
4. What do you do when you go to buy pork?

**Probes**

1. where do you buy,
2. what do you do when you reach the place of buying?
3. what do you look for in the meat before buying?
4. What do you do if you don’t like the meat?
5. Do you prepare pork in the house or you eat out?

**Probes**

1. How do you prepare your pork?
2. What are the effects of eating poorly cooked pork?
3. where do you buy the pork?
4. do you check for cysts when buying?
5. Do you deworm yourselves and family members? (**probe for drug used, where they get it from**
6. What do you think will make it easy for people to practice the control measures?

**Workshop guide for meat inspectors/animal health assistants**

1. What is Taenia solium?

**Probes**

1. perceived causes,
2. how is it transmitted to pigs and from pigs to humans?
3. how it manifests in humans and pigs,
4. what can a mother/farmer/butcher do to break the cycle?
5. How can we control these 3 diseases (allow them brainstorm and use the poster to show possible control options (the 6 stages to break the cycle)? Give an overview of the control options which can be done at community level and discuss their roles as outline below)
6. Mass drug administration (MDA) in humans -PZQ (In general population or in School Age Children (SAC) – when children or adults are given deworming tablets.

**Probes**

What can motivate you to participate in this option (deworming for other soil transmitted helminths?)

1. Vaccine + oxfendazole (OXF) in pigs-*(vaccine-TSOL18 kills cysts in pigs, OXF kills T. solium cysts and kills other pig worms)*

**Probes**

1. will farmers be willing to pay for the vaccine?
2. What can motivate you to adopt this option?
3. Is worm infection in pigs a problem in this area?
4. Will use of a drug that kills both pig worms and *T. solium* in pigs be suitable for you? why?
5. Health education – on pig husbandry and household and community hygiene – use the T. solium poster (why confining pigs and feeding them clean feed is important, why use of toilet and washing hands is important
6. Give overall results from the cross-sectional study and highlight the issues with the diagnostic method in terms of sensitivity and specificity (prevalence, risk factors).
7. Have you ever received any training on detection of PCC **(probe for when by who, what were you trained on)?**
8. Have ever condemned pig carcass due to PCC?

**Probes:**

1. Which are the common reasons for condemning
2. what happens to the carcass?
3. who makes sure it disposed?
4. What do you do when you find cysts in pork?

**probes**

1. what advise you give to the butcher, do you condemn?
2. If you condemn, how is the reaction by the trader/butcher/farmer
3. How do they recover the cost?
4. any other markets for the condemned pig,
5. do they fight back?
6. How do you ensure they don’t get back to you?
7. Is there enough support from the government to protect you and how can it be improved?
8. Are there laws or acts which guide your work (which are they, what does the law say?)
9. How is the compliance of farmers on pig husbandry practices (housing, feeding and treatment/deworming)?
10. In your view what makes farmers not to adopt improved pig husbandry practices (Housing/feeding/deworming or treating)
11. Do you keep records when you treat pigs?

**Probes**

1. what do you include in the record?
2. how often do farmers call you to treat their pigs?
3. what are the common issues you handle?
4. What do you think will make it easy for people to practice the control measures?

**Workshop guide for community human health assistants**

1. What is Taenia solium?

**Probes**

1. perceived causes,
2. how is it transmitted to pigs and from pigs to humans?
3. how it manifests in humans and pigs,
4. what can a mother/farmer/butcher do to break the cycle?
5. How can we control these 3 diseases (allow them brainstorm and use the poster to show possible control options (the 6 stages to break the cycle)? Give an overview of the control options which can be done at community level and discuss their roles as outline below)
6. Mass drug administration (MDA) in humans -PZQ (In general population or in School Age Children (SAC) – when children or adults are given deworming tablets.

**Probes**

What can motivate you to participate in this option (deworming for other soil transmitted helminths?)

1. Vaccine + oxfendazole (OXF) in pigs-*(vaccine-TSOL18 kills cysts in pigs, OXF kills T. solium cysts and kills other pig worms)*

**Probes**

1. will farmers be willing to pay for the vaccine?
2. What can motivate you to adopt this option?
3. Is worm infection in pigs a problem in this area?
4. Will use of a drug that kills both pig worms and *T. solium* in pigs be suitable for you? why?
5. Health education – on pig husbandry and household and community hygiene – use the T. solium poster (why confining pigs and feeding them clean feed is important, why use of toilet and washing hands is important
6. Give overall results from the cross-sectional study and highlight the issues with the diagnostic method in terms of sensitivity and specificity (prevalence, risk factors). Brainstorm on the meaning of the results and how they can be used.
7. How many households have toilets in your area?

**Probes**

1. percentage of households with toilets,
2. are the toilets well-constructed?
3. What type of toilet are there in the subcounty?
4. what is the role of men and women in construction of toilets?
5. Are there programs by government or NGOs supporting construction of toilets or hygienic infrastructure?
6. Where do households without toilets relieve themselves?

**Probes**

1. households with children below five years, elderly, men and women
2. why do they not use toilets?
3. How do households dispose the waste?
4. Are there households with toilets and do not use them? **Why?**
5. What are the constraints to toilet construction? (**Probe to know how they affect men and women, what can be done to increase construction and use of toilets)**
6. Do the local marketplaces have a public toilet (**Probe to know where people relieve themselves while in the local markets, during village gathering/meetings)**
7. What programmes do you have which support deworming of households and children?
8. How do you support people with epilepsy (diagnosis, medication, community education to avoid stigmatization)?
9. What are the types or causes of epilepsy in the community? How many people have epilepsy in the community?
10. What do you think will make it easy for people to practice the control measures?

**Workshop guide for traders (they operate as pig traders but also have a pork joint/butchery)**

1. What is *Taenia solium*?

**Probes**

1. perceived causes,
2. how is it transmitted to pigs and from pigs to humans?
3. how it manifests in humans and pigs?
4. what can a mother/farmer/butcher do to break the cycle? - use the T. solium poster to explain the causal and impact pathways of the three diseases)
5. Give overall results from the cross-sectional study and highlight the issues with the diagnostic method in terms of sensitivity and specificity (prevalence, risk factors).
6. How can we control these 3 diseases (allow them brainstorm and use the poster to show possible control options (the 6 stages to break the cycle)? What do they think is their role in the steps? Give an overview of the control options which can be done at community level and discuss their roles as outline below)
7. Mass drug administration (MDA) in humans -PZQ (In general population or in School Age Children (SAC) – when children or adults are given deworming tablets. What can motivate you to participate in this option (deworming for other soil transmitted helminths?)
8. Vaccine + oxfendazole (OXF) in pigs-*(vaccine-TSOL18 kills cysts in pigs, OXF kills T. solium cysts and kills other pig worms)*

***Probes***

- will you be willing to pay for the vaccine?
- What can motivate you to adopt this option?
- Is worm infection in pigs a problem in this area?
- Will use of a drug that kills both pig worms and *T. solium* in pigs be suitable for you? why?

1. Health education – on pig husbandry and household and community hygiene – use the T. solium poster (why confining pigs and feeding them clean feed is important, why use of toilet and washing hands is important
2. What qualities do you look for when buying pigs? (What health criteria/health checks do you use)
3. Do you inspect for diseases (what diseases do you look for? How do you check whether the pig is sick? How do you inspect for PCC or cysts in pigs)
4. What will make it easier for you to do the health checks?

**Probes**

1. provision of new technologies for testing at the farm,
2. prove that the pigs have been treated/vaccinated e.g. vaccination certificate)
3. What do you do when you find cysts in pork?

**Probes**

1. do you still buy?
2. Do you buy at the same price?
3. Where do you take that pig after buying?
4. Have you ever received any training on detection of cysts (probe for when by who, what were you trained on)?
5. If you slaughter pigs, where do you slaughter from (probe for private slaughter slab, private commercial slaughter slab, government slaughter slab/house).
6. What are your views about meat inspection by government?

**Probes**

1. is it important to be done?
2. Who does the inspection?
3. What happens if they are not available to inspect?
4. If the carcass does not pass the inspection,
5. what happens?
6. Are there laws or acts which prevent you from selling/trading in sick pigs or movement of pigs **(which are they, what does the law say? What does the government require from you)?**
7. What do you think will make it easy for people to practice the control measures? (include group ranking exercise

**Workshop guide for community leaders (LC1 chairman/village leader)**

1. What is Taenia solium?

**Probes**

1. perceived causes,
2. how is it transmitted to pigs and from pigs to humans?
3. how it manifests in humans and pigs?
4. what can a mother/farmer/butcher do to break the cycle? - use the T. solium poster to explain the causal and impact pathways of the three diseases)
5. Give overall results from the cross-sectional study and highlight the issues with the diagnostic method in terms of sensitivity and specificity (prevalence, risk factors). **Brainstorm on the meaning of the results and how they can be used.**
6. How can we control these 3 diseases (allow them brainstorm and use the poster to show possible control options (the 6 stages to break the cycle)? Give an overview of the control options which can be done at community level and discuss their roles as outline below)
7. Mass drug administration (MDA) in humans -PZQ (In general population or in School Age Children (SAC) – when children or adults are given deworming tablets in mass. **What can motivate you to participate in this option (deworming for other soil transmitted helminths?)**
8. Vaccine + oxfendazole (OXF) in pigs-*(vaccine-TSOL18 kills cysts in pigs, OXF kills T. solium cysts and kills other pig worms)*

***Probes***

- will you be willing to pay for the vaccine?
- What can motivate you to adopt this option?
- Is worm infection in pigs a problem in this area?
- Will use of a drug that kills both pig worms and *T. solium* in pigs be suitable for you? why?

1. Health education – on pig husbandry and household and community hygiene – use the T. solium poster (why confining pigs and feeding them clean feed is important, why use of toilet and washing hands is important
2. How many households have toilets in your area

**Probes**

1. percentage of households with toilets,
2. are the toilets well-constructed?
3. what is the role of men and women in construction of toilets?
4. Are there programs by government or NGOs supporting construction of toilets or hygienic infrastructure?)
5. Where do households without toilets relieve themselves? **(Probe for households with children below five years, elderly, men and women)**
6. Where do households without toilets relieve themselves? **(Probe for households with children below five years, elderly, men and women) why do they not use toilets? How do households dispose the waste?**
7. Do the local marketplaces have a public toilet **(Probe to know where people relieve themselves while in the local markets, during village gathering/meetings)**
8. What is your role in the control of T. solium infections? (probe for role in promoting/ enforcing sanitation, community deworming programmes, pig husbandry e.g. preventing pigs from roaming, toilet use)
9. Has any of the above strategies been implemented in your village (probe for the scale/period, implementer and participation of the stakeholder or actor)

**Key informant guide for district level governmental and non-governmental actors (DVO, DHO, IOWA, NARO, HOCADEO, Private Vet)**

1. Use the *T. solium* poster to explain the parasite and the 3 diseases – Taeniasis, Porcine cysticercosis and Neurocysticercosis (probe first on what they understand about *T. solium* transmission)
2. Give overall results from the cross-sectional study and highlight the issues with the diagnostic method in terms of sensitivity and specificity (prevalence, risk factors). Brainstorm on the meaning of the results and how they can be used.
3. How can we control these 3 diseases (allow them brainstorm and use the poster to show possible control options (the 6 stages to break the cycle)? Give an overview of the control options which can be done at community level and discuss their roles as outline below)
4. Mass drug administration (MDA) in humans -PZQ (In general population or in School Age Children (SAC) – when children or adults are given deworming tablets. What can motivate you to participate in this option (deworming for other soil transmitted helminths?)
5. Vaccine + oxfendazole (OXF) in pigs-*(vaccine-TSOL18 kills cysts in pigs, OXF kills T. solium cysts and kills other pig worms)* (will you be willing to pay for the vaccine? What can motivate you to adopt this option? Is worm infection in pigs a problem in this area? Will use of a drug that kills both pig worms and *T. solium* in pigs be suitable for you? why?
6. Health education – on pig husbandry and household and community hygiene – use the T. solium poster (why confining pigs and feeding them clean feed is important, why use of toilet and washing hands is important
7. Has any of the above strategies been implemented in your village (probe for the scale/period, implementer and participation of the stakeholder or actor)
8. Are there regulation which support the control of T. solium (what are these laws, do you play a role in enforcement?)
9. What activities do you do to support work zoonotic diseases (including *T. solium*) in your area of operation?
10. What is your role in the control of *T. solium* infections?
11. What is your role in creating support structures (allocating staff to the control initiatives, lobbying and procuring drugs) to support success of the interventions?
12. Do you work with other agencies or arms of government/NGOs/private sector (which are they, what type of data do you share?
13. What motivation will support your staff to participate in control activities (what are some the things which could make staff unwilling to participate)
14. What do you think are some of the barriers to adopting the control packages discussed (MDA, Vaccine + OXF and Health education)? How can the barriers be overcome?
